# Spine system changes in soldiers after load carriage training in a plateau environment: a prediction model research

**DOI:** 10.1186/s40779-020-00293-1

**Published:** 2020-12-21

**Authors:** Hao Qu, Ling-Jia Yu, Ju-Tai Wu, Gang Liu, Sheng-Hui Liu, Peng Teng, Li Ding, Yu Zhao

**Affiliations:** 1 Department of Orthopaedics, Peking Union Medical College Hospital, Chinese Academy of Medical Sciences and Peking Union Medical College, Beijing, 100730 China; 2 Department of Orthopaedics, Beijing Friendship Hospital, Beijing, 100050 China; 3 Department of Orthopaedics, the Affiliated Hospital of Xuzhou Medical University, Xuzhou, 221006 Jiangsu China; 4 Department of Radiology, Qinghai Red Cross Hospital, Xining, 810000 Qinghai China; 5 School of Biological Science and Medical Engineering, Beihang University, Beijing, 100191 China; 6 National Laboratory of Human Factors Engineering, China Astronaut Research and Training Center, Beijing, 100094 China

**Keywords:** Spine, Load carriage, Paraspinal muscle, Intervertebral disc, Prediction model

## Abstract

**Background:**

Low back pain is the most common spinal disorder among soldiers, and load carriage training (LCT) is considered the main cause. We aimed to investigate changes in the spine system of soldiers after LCT at high altitudes and the change trend of the lumbar spine and surrounding soft tissues under different load conditions.

**Methods:**

Magnetic resonance imaging scans of the lumbar spines of nine soldiers from plateau troops were collected and processed. We used ImageJ and Surgimap software to analyze changes in the lumbar paraspinal muscles, intervertebral discs (IVDs), intervertebral foramina, and curvature. Furthermore, the multiple linear regression equation for spine injury owing to LCT at high altitudes was established as the mathematical prediction model using SPSS Statistics version 23.0 software.

**Results:**

In the paraspinal muscles, the cross-sectional area (CSA) increased significantly from 9126.4 ± 691.6 mm^2^ to 9862.7 ± 456.4 mm^2^, and the functional CSA (FCSA) increased significantly from 8089.6 ± 707.7 mm^2^ to 8747.9 ± 426.2 mm^2^ after LCT (*P* < 0.05); however, the FCSA/CSA was not significantly different. Regarding IVD, the total lumbar spine showed a decreasing trend after LCT with a significant difference (*P* < 0.05). Regarding the lumbar intervertebral foramen, the percentage of the effective intervertebral foraminal area of L_3_/_4_ significantly decreased from 91.6 ± 2.0 to 88.1% ± 2.9% (*P* < 0.05). For curvature, the lumbosacral angle after LCT (32.4° ± 6.8°) was significantly higher (*P* < 0.05) than that before LCT (26.6° ± 5.3°), while the lumbar lordosis angle increased significantly from 24.0° ± 7.1° to 30.6° ± 7.4° (*P* < 0.05). The linear regression equation of the change rate, △FCSA% = − 0.718 + 23.085 × load weight, was successfully established as a prediction model of spinal injury after LCT at high altitudes.

**Conclusion:**

The spinal system encountered increased muscle volume, muscle congestion, tissue edema, IVD compression, decreased effective intervertebral foramen area, and increased lumbar curvature after LCT, which revealed important pathophysiological mechanisms of lumbar spinal disorders in soldiers following short-term and high-load weight training. The injury prediction model of the spinal system confirmed that a load weight < 60% of soldiers’ weight cannot cause acute pathological injury after short-term LCT, providing a reference supporting the formulation of the load weight standard for LCT.

## Background

Chronic low back pain is the most common musculoskeletal disorder among soldiers. According to statistics, > 50% of soldiers have experienced low back pain during service [1], and the prevalence rate of low back pain in new recruits during basic training reaches 30.3% [2]. Chronic low back pain is also the most common illness in the United States (US) military that seriously affects the normal training of soldiers and reduces the service life and quality of active-duty soldiers [3]. At present, commonly considered main risk factors of low back pain in soldiers during military training include weight, training environment, and psychological and organizational management factors. Among them, excessive load carriage training (LCT) is considered the main cause of back pain and injuries among soldiers [4].

In LCT, a soldier carries a certain weight of marching combat equipment according to the requirements of the mission, battlefield environment, weather conditions, and other factors when performing training or combat tasks. Then, the soldiers carry different weights across various terrains to complete various operational tasks to a high standard. A previous study described that under general temperature conditions when the marching speed is 5 km/h, the maximum allowable load weight for soldiers is 25 kg, and the most acceptable weight is 20 kg [5]. However, during actual training or combat, soldiers always carry heavier loads than the current standards set by the military. For example, the US military standard requires 25 kg as the maximum load weight of field soldiers in marches; however, during the Iraq war, the actual load weight of US soldiers was approximately 35 kg, which could reach 42–75 kg when soldiers were approaching the enemy [6].

At present, “plateau adaptive training” is an important training method for endurance and perseverance. In a high-altitude environment (> 500 m above sea level), some factors such as low partial pressure of air and oxygen caused by high altitude and dry and cold climate reduce the alveolar oxygen partial pressure, blood oxygen saturation, energy consumption, and metabolic rate of soldiers during training. Thus, both the maximum and acceptable load weights are reduced accordingly. Yin et al. [7] suggested that at a constant marching speed of 4 km/h, when the altitude of the training environment increased from 3700 m to 4300 m, the appropriate load weight of soldiers should be reduced from 20.0 kg to 11.5 kg, and the reduction rate is close to 50%. To deal with the adverse effects of load weight reduction on military training in the plateau environment, at present, the military mainly increases the maximum load weight of soldiers to improve their aerobic endurance so as to strengthen the plateau LCT. However, this often causes an overload on the spine system, accelerates strain on the paraspinal muscles and intervertebral discs (IVDs), and even leads to changes in the spine curvature and spinal cord compression [8, 9]. Hrubec et al. [10] reported that 1095 soldiers had been treated for low back pain after World War II. Recent studies have found that soldiers have a higher risk of lumbar disc herniation than ordinary people. Moreover, some studies have reported that as the load weight of soldiers increases, the forward tilt angle of the lumbar spine also increases, which in turn affects the balance and increases the risk for a spinal disorder [11].

To the best of our knowledge, there is a lack of standards for plateau LCT. Existing military studies on plateau training have focused more on respiration, sleep, or psychological problems of soldiers [12], whereas little attention has been paid to changes in the spine system of soldiers who carry varying load weights during plateau training. Therefore, we aimed to investigate changes in the spine system of soldiers during plateau LCT and the trend of the lumbar spine change under different load conditions. By obtaining radiographic lumbar images of soldiers, we established a prediction model for lumbar spine injury after LCT at high altitude.

## Methods

### Experimental design

Nine soldiers from combat troops in Xining City, Qinghai Province (2443 m above sea level), volunteered for this study. All subjects were of Han nationality with the following characteristics: age, 18–31 years; weight, 60–80 kg; height, 168–182 cm; and service life, 1–10 years on the plateau (Table 1). The training scores and muscle strength of these soldiers were up to the excellent standard. Since the interventions in this study may lead to injury, we ensured that soldiers were trained safely. With the limited military training schedule and secrecy requirements, we could not directly obtain image data of the spines from an adequate number of soldiers. To simulate the LCT of plateau soldiers with different load weights and time, we used a uniform experimental design (Table 2). The load weight (percentage of soldiers’ weight) was Factor 1, which included 20, 40, and 60% of the soldiers’ weight, and the load time was Factor 2, which included 20 min, 40 min, and 60 min. The subjects were grouped by draw lots as a completely randomized grouping. In this experimental design, one participant was assigned to one test condition (i.e., subject 1 had a 40-min LCT with 20% load weight).

**Table 1:** Basic information of the subjects

No.	Age (year)	LMS (year)	Height (cm)	Weight (kg)	Past medical history
1	24	1	171	78	None
2	31	10	170	74	None
3	27	9	171	72	None
4	23	1	173	63	None
5	30	10	170	76	None
6	20	2	168	63	None
7	18	1	170	60	None
8	21	3	177	65	None
9	26	7	172	80	Fracture of right hand

*LMS* Length of military service

**Table 2:** Uniform experimental design of plateau LCT

Factor 2 (Load time)	Factor 1 (Load weight)		
	20%	40%	60%
20 min	No. 4	No. 3	No. 6
40 min	No. 5	No. 9	No. 1
60 min	No. 8	No. 7	No. 2

*LCT* Load carriage training

This study was approved by the ethics committee of the Qinghai Red Cross Hospital (KY-2020-15), and each subject provided written informed consent.

### Research equipment

Lumbar spine magnetic resonance imaging (MRI) was performed before and after the LCT by using the GE Signa Explorer 1.5 T MRI system at the Qinghai Red Cross Hospital. As for the examination time, some studies have shown variations on the lumbar flexion range, lumbar IVD height, and other biomechanical characteristics at different times of the day, known as the diurnal effect [13, 14]. To avoid this effect, all soldiers were trained uniformly in the morning and MRI was completed within 30 min after LCT.

**Fig. 1:**
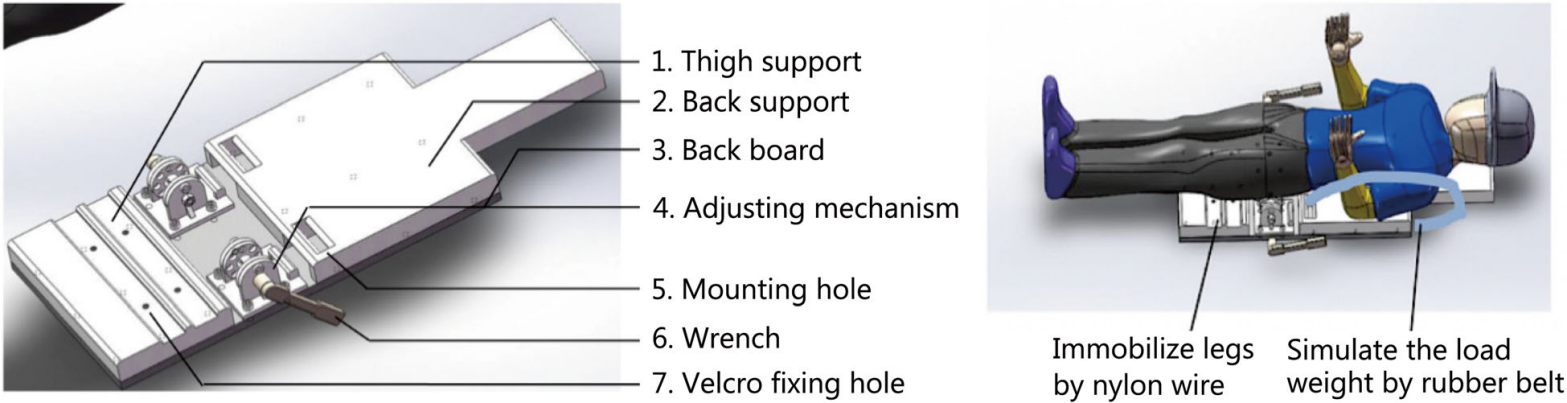
Pressure device for simulating the soldier back loaded in the supine position

**Fig. 2:**
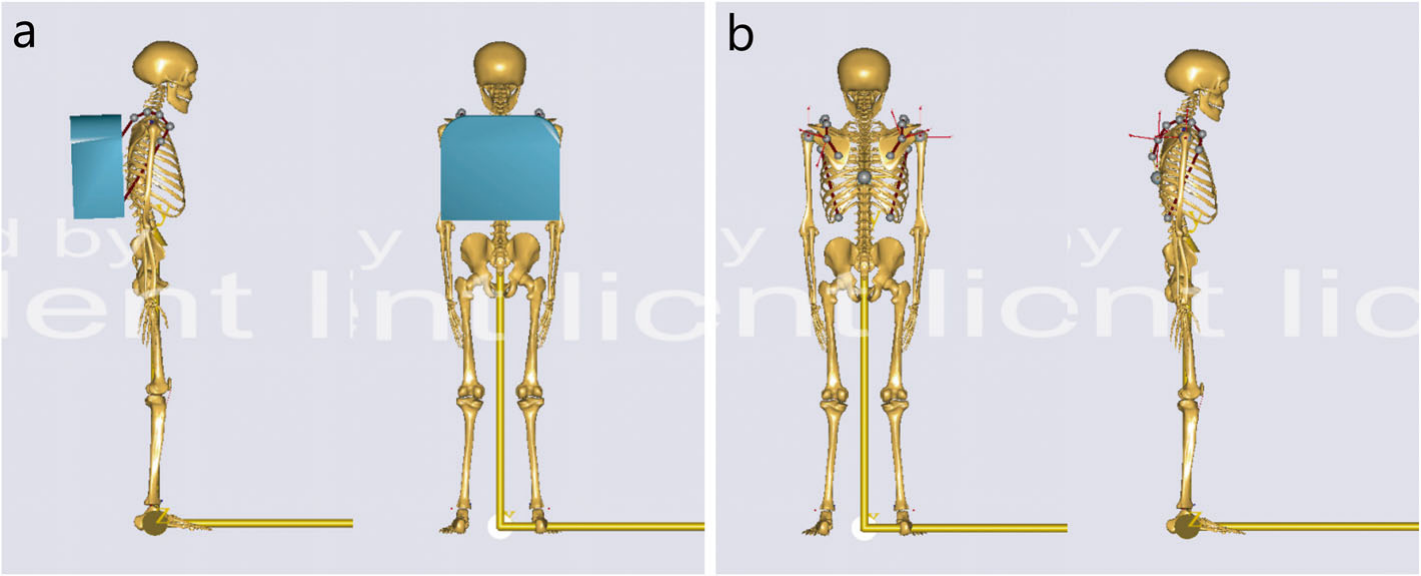
Simulation mechanics analysis of the human body with load weight. **a** The simulation model with load weight by carrying a backpack. **b** The simulation model with load weight using the pressure device

Because of the limited supine position during training, we could not directly obtain actual spine images of soldiers who were standing with different load weights after training. Therefore, a pressure device was designed in advance for this study to simulate the soldiers’ loaded backs when they were in the lying position (Fig. 1). Detailed steps were as follows: we used nylon wire through the Velcro fixing hole (7) to immobilize the legs, and a rubber belt was used to link the subject’s shoulder to the adjusting mechanism (4) through the mounting hole (5) (Fig. 1). The adjusting mechanism was then used to adjust the length of the belt to simulate the load weight on the back. This device is made of non-metallic materials and can be used during MRI to obtain images of the spine under different load weights. The effectiveness of this device was verified by simulation mechanics analysis using AnyBody Modeling System, version 4.0 software (AnyBody Technology, Aalborg, Denmark), that is, it can simulate the loaded state of the human body in the standing position (Fig. 2).

According to the experimental design, to perform MRI before LCT, the subjects were lying supine on the device without support on the lumbar spine. Then, each subject carried a backpack weighting a percentage of their weight during LCT. After LCT, the subject was fixed to the device in the same posture, and the spring dynamometer was attached to one end of the rubber belt to apply a tension pressure, which is equal to the subject’s backpack weight, as the load weight. Finally, we affixed the rubber belt and performed MRI.

### Research indicators

#### Lumbar paraspinal muscle

**Fig. 3:**
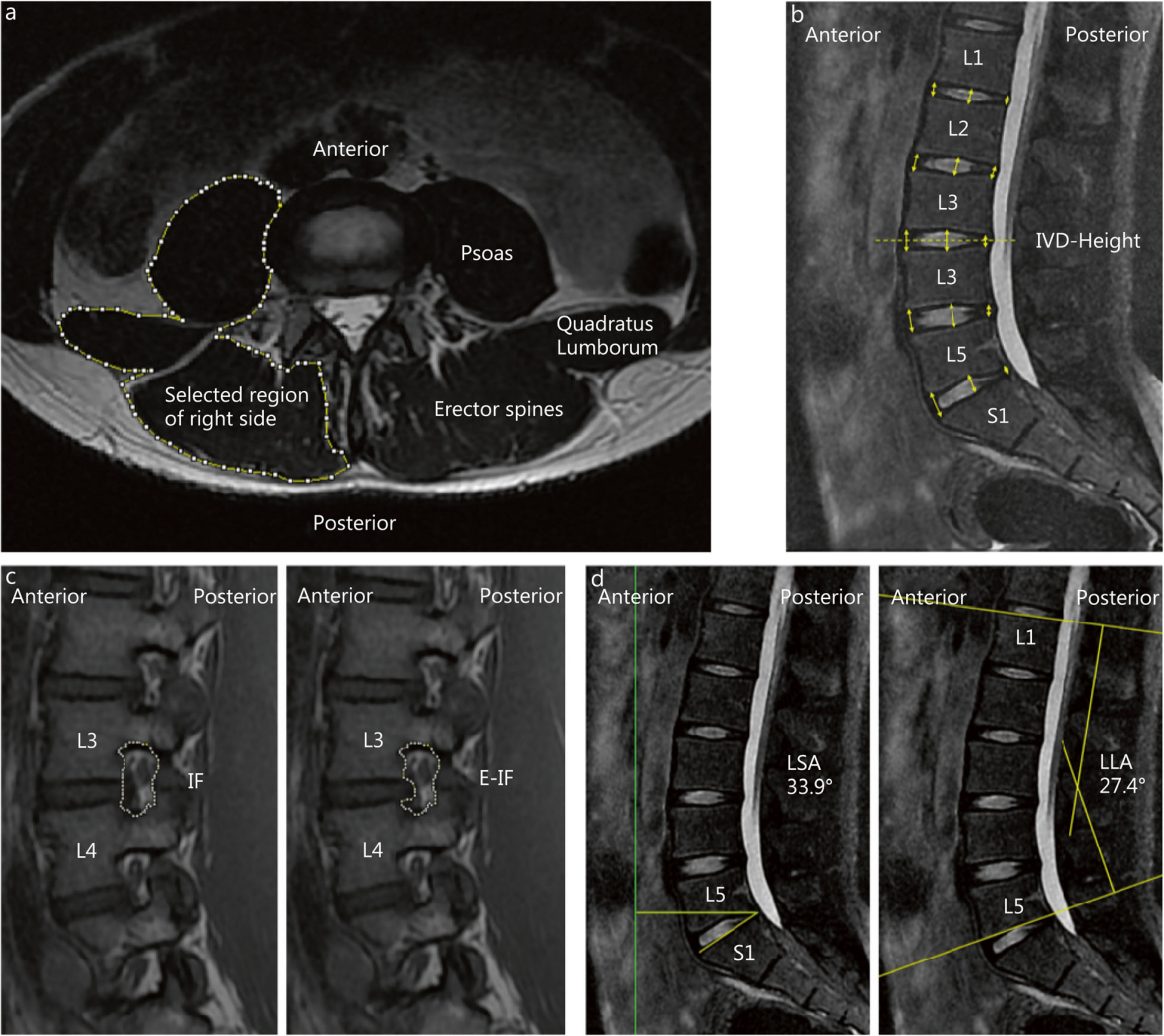
Research indicators of the lumbar spine and measurement methods. **a** The location of the lumbar paraspinal muscles identified for the cross-sectional area (CSA) and functional cross-sectional area (FCSA) at the L_3_/_4_ level. **b** The measurement for intervertebral discs (IVD) height (anterior, middle, and posterior) from L_1_ to S_1_. **c** The measurement for the intervertebral foramen area (IF) and the effective intervertebral foraminal area (E-IF) in L_3_/_4_ segments. **d** The measurement for the lumbosacral angle (LSA) and the lumbar lordosis angle (LLA)

Changes in the paraspinal muscles can be detected based on the cross-sectional area (CSA), fat content, and proportions of fast-twitch fibers [15]. The CSA image was obtained from T_2_-weighted MRI scans. Because the boundary of the paraspinal muscle at the L_3/4_ vertebral level can be clearly identified, we used ImageJ version 1.51 software (National Institute of Mental Health, Bethesda, MD, USA) to manually mark the contour of the paraspinal muscle at this level and then summed the CSAs of the following paraspinal muscles: bilateral erector spinae, bilateral psoas major, and bilateral quadratus lumborum (Fig. 3). The CSA was used to quantify the level of hypertrophy or atrophy of the lumbar paraspinal muscles [16, 17].

At the same time, Parkkola et al. [18] confirmed that when the paraspinal muscles undergo physiological changes, the CSA may not change correspondingly because some tissue fluids, fats, or other fibrous tissues fill the original locations of the muscle fibers. Ranson et al. [19] reported that the gray value threshold of the muscle fibers in T_2_-weighted MRI scans was approximately 0–120 pixels (30–240 pixels for the bones and 120–660 pixels for the fats). Based on this fact, we obtained the gray values of images of the paraspinal muscles before and after LCT. Then, to determine the functional CSA (FCSA) of the paraspinal muscle, the threshold analysis method was used to select the image area in the CSA within the gray value threshold of 0–120 pixels. FCSA was used to represent the total content of lean muscle fibers within the boundaries of the lumbar paraspinal muscle fascia to measure changes in the muscle fibers. A related control study by Fortin et al. [20] showed that this image measurement method has certain reliability and repeatability regardless of whether it is performed by one or more people, and the intra-group correlation coefficient is 0.99.

#### Lumbar IVD height

We measured the height of the lumbar IVD on T_2_-weighted MRI scans at the midsagittal plane with a slice thickness of 3.5 mm. According to the method of Dabbs, we measured the disc height at the anterior, middle, and posterior sections at L_1_/_2_, L_2_/_3_, L_3_/_4_, L_4_/_5_, and L_5_/S_1_ levels before and after LCT [21] (Fig. 3). The disc height was defined as the average of three height values at a given level. Finally, the difference in IVD height before and after LCT and the change in the total IVD height were compared.

#### Lumbar intervertebral foramen

We measured the lumbar intervertebral foramen on T_2_-weighted MRI scans at the sagittal plane. Clinical investigations showed that the intervertebral foramina in the L_3_/_4_ and L_4_/_5_ segments are located at the physiological bend of the lumbar spine. Due to stress concentration during weight loading, the annulus fibrosus is prone to damage under the action of external forces; thus, the degeneration in these segments is more severe than in other segments [22, 23]. Therefore, we used the measurement method similar to CSA: we artificially marked the contour of the L_3_/_4_ and L_4_/_5_ intervertebral foramina on both the left and right sides (the upper, lower, and outer borders of the intervertebral foramina are bone boundaries, and the inner boundary is the line connecting the upper and lower vertebral bodies, Fig. 3) and measured this area as the intervertebral foraminal (IF) area. Then, we kept the upper, lower, and outer borders unchanged, marked the inner border again as the IVD boundary (Fig. 3), and measured this area as the effective IF (E-IF) area. Finally, the ratio of these two areas (E-IF/IF) was defined as the percentage of the effective IF area (E-IF%), which represented the effective space rate for nerve root activity in the lumbar intervertebral foramen.

#### Lumbar curvature

We measured the lumbosacral angle (LSA) and lumbar lordosis in the midsagittal T_2_-weighted image using Surgimap version 2.2.15 software (Nemaris, NY, USA) to evaluate the lumbar curvature. The LSA was measured as the angle of intersection between the extension of the upper margin of the sacrum and the horizontal line [24]. Because the lumbosacral region is the joint point of stress when the body is upright or loaded, this angle can indicate the stability of the lumbar spine. The lumbar lordosis angle (LLA) was measured using Cobb’s method, which measures the angle between the L_1_ vertebral upper endplate and L_5_ vertebral lower endplate extension line (Fig. 3) [25].

### Questionnaire survey

The subjective questionnaire survey was performed and analyzed on nine participants. The questionnaire included basic information, degree of low back pain, degree of fatigue, and other physical symptoms. The low back pain level was rated by using an 11-point numerical rating scale (NRS) (scored from 0 to 10, with 0 as painless, 1–3 as mild pain, 4–6 as moderate pain, 7–9 as severe pain, 10 as twinge), and the degree of fatigue was self-rated from 0 to 10 (0 as energetic, 10 as exhausted), which means that the higher the score, the more the physical exertion [26, 27]. All questionnaires were completed independently by the subjects before and after LCT.

### Statistical analysis

All data of each research index were analyzed using SPSS Statistics version 23.0 (IBM Corp., Armonk, NY, USA). First, the mean value analysis was used to evaluate the characteristics of changes before and after LCT. Second, significance analysis (*P* < 0.05) and simulation regression analysis were performed for the change rates (△Index%) of different indexes (△LCT% = (post LCT value – pre LCT value)/pre LCT value). Detailed steps are as follows: 1) Shapiro-Wilk tests were used to check data normality (*P* > 0.05). 2) Paired sample *t*-tests were used to compare significant differences between each index before and after LCT (*P* < 0.05). 3) The main effect variance analysis method was used to analyze whether there were significant differences in the changes in various indicators between groups with different load weights and between those with different load times (*P* < 0.05). 4) Multiple comparison analysis was used to further compare differences in the index changes in the same load weight group or the same load time group (*P* < 0.05). 4) A specific index was selected to fit the multiple linear regression equation: the change rate before and after LCT = b1 + b2 × load weight + b3 × load time, which we used as a mathematical prediction model.

## Results

### Lumbar paraspinal muscle indexes

The CSA increased from 9126.371 ± 691.614 mm^2^ to 9862.655 ± 456.416 mm^2^ after LCT, with an average increase of 8.07%. The paired *t*-test showed that the CSA was significantly different before and after LCT (*t =* − 6.368, *P* = 0.0002, Table 3). The average gray value of the paraspinal muscle was reduced from 80.396 ± 17.522 to 55.009 ± 10.641 after LCT, with an average decrease of 31.58%. The paired *t*-test showed significant differences before and after LCT (*t =* 5.968, *P* = 0.0003, Table 3). The FCSA before LCT was 8089.556 ± 707.739 mm^2^, and it increased to 8747.917 ± 426.245 mm^2^ after LCT, with an average increase of 8.14%. The paired *t*-test showed that the FCSA was significantly different before and after LCT (*t =* − 5.348, *P* = 0.0007, Table 3).

**Table 3:** Statistical analysis results of lumbar spine indexes

Item	Pre LCT	Post LCT	Average rate of change (%)	*t*	*P*
CSA (mm^2^)	9126.371 ± 691.614	9862.655 ± 456.416	8.07	−6.368	0.0002
FCSA (mm^2^)	8089.556 ± 707.739	8747.917 ± 426.245	8.14	−5.348	0.0007
Gray value	80.396 ± 17.522	55.009 ± 10.641	−31.58	5.968	0.0003
FCSA/CSA	0.886 ± 0.032	0.887 ± 0.024	0.12	− 0.169	0.8703
L_1_/_2_ IVD (pixel)	14.486 ± 1.854	13.790 ± 1.933	− 4.80	3.308	0.0107
L_2_/_3_ IVD (pixel)	16.781 ± 2.559	16.671 ± 1.918	−0.66	0.455	0.6610
L_3_/_4_ IVD (pixel)	18.693 ± 2.105	18.120 ± 2.047	−3.07	2.278	0.0522
L_4_/_5_ IVD (pixel)	20.018 ± 2.169	19.290 ± 1.751	−3.64	3.509	0.0080
L_5_/S_1_ IVD (pixel)	17.266 ± 2.359	16.888 ± 2.815	−2.19	0.889	0.3999
L_1_-S_1_ IVD (pixel)	87.243 ± 8.943	84.759 ± 7.620	− 2.85	2.971	0.0180
L_3_/_4_ E-IF%	91.6 ± 2.0	88.1 ± 2.9	−3.80	8.214	0.0004
L_4_/_5_ E-IF%	89.6 ± 1.9	88.2 ± 1.7	−1.66	2.553	0.0511
LSA(°)	26.622 ± 5.303	32.422 ± 6.816	21.79	−4.911	0.0012
LLA(°)	24.011 ± 7.117	30.644 ± 7.415	27.63	− 4.851	0.0013

*LCT* Load carriage training, *CSA* Cross-sectional area, *FCSA* Functional cross-sectional area, *IVD* Intervertebral discs, *E-IF%* Percentage of effective intervertebral foramen area, *LSA* Lumbosacral angle, *LLA* Lumbar lordosis angle

The FCSA/CSA changed from 0.886 ± 0.032 to 0.887 ± 0.024 after LCT, with an average change of 0.12%. No significant difference was found before and after LCT (*t =* −0.169, *P* = 0.8703). After the analysis of the main effect variance, no significant difference in the parameters of the lumbar paraspinal muscle between the two groups was found, regardless of whether they were grouped by different load weight or load time (Table 4).

**Table 4:** Results of the main effect variance analysis

Item	Load time					Load weight				
	DEVSQ	Df	Mean square	*F*	*P*	DEVSQ	Df	Mean square	*F*	*P*
CSA	12.258	2	6.129	0.243	0.7915	86.341	2	43.171	3.359	0.1050
Gray value	660.432	2	330.216	2.925	0.1298	165.991	2	82.995	0.425	0.6721
FCSA	8.102	2	4.051	0.114	0.8940	128.419	2	64.209	4.159	0.0736
FCSA/CSA	3.249	2	1.624	0.283	0.7630	13.060	2	6.530	1.591	0.2791
L_1_/_2_ IVD	10.450	2	5.225	0.189	0.8322	32.111	2	16.055	0.670	0.5463
L_2_/_3_ IVD	36.934	2	18.467	1.072	0.3999	72.082	2	36.041	3.171	0.1149
L_3_/_4_ IVD	57.379	2	28.690	3.193	0.1137	3.654	2	1.827	0.102	0.9047
L_4_/_5_ IVD	4.001	2	2.001	0.209	0.8169	15.676	2	7.838	1.029	0.4130
L_5_/S_1_ IVD	168.697	2	84.349	1.737	0.2539	29.461	2	14.731	0.205	0.8199
L_1_-S_1_ IVD	19.978	2	9.989	2.343	0.1770	7.759	2	3.880	0.616	0.5712
L_3_/_4_ E-IF%	0.000	2	0.000	1.462	0.5050	0.000	2	0.000	1.573	0.4910
L_4_/_5_ E-IF%	5.789	2	2.894	0.636	0.6640	0.560	2	0.280	0.062	0.9441
LSA	98.999	2	49.500	0.217	0.8111	1075.995	2	537.998	8.228	0.0191
LLA	1822.127	2	911.064	1.922	0.2264	1117.132	2	558.566	0.944	0.4400

*DEVSQ* Deviation sum of squares, *Df* Degrees of freedom, *CSA* Cross-sectional area, *FCSA* Functional cross-sectional area, *IVD* Intervertebral discs, *E-IF%* Percentage of effective intervertebral foramen area, *LSA* Lumbosacral angle, *LLA* Lumbar lordosis angle

### Lumbar IVD height indexes

The disc height at each level of L_1_-S_1_ and the total height showed a consistent decreasing trend after LCT. The total disc height decreased from 87.243 ± 8.943 pixels to 84.759 ± 7.620 pixels, with an average decrease of 2.85%. The paired *t*-test showed a significant difference in the total disc height before and after LCT (*t =* 2.971, *P* = 0.0180, Table 3). Moreover, a significant difference was found in the analysis results of the disc height at L_1_/_2_ (*t =* 3.308, *P* = 0.0107) and L_4_/_5_ level (*t =* 3.509, *P* = 0.0080). A related study reported that the standard deviations of this measurement method between observers and within observers are 0.2 mm and 0.3 mm, respectively [28]. After the analysis of the main effect variance, no significant differences in the total height or each height at different levels of the lumbar IVD were found between the two groups, regardless of whether they were grouped by load weight or load time (Table 4).

### Lumbar IF indexes

The E-IF% of L_3_/_4_ decreased from 91.6% ± 2.0 to 88.1% ± 2.9% after LCT, with an average reduction of 3.80%. The paired *t*-test indicated a significant difference in E-IF% of L_3_/_4_ (*t =* 8.214, *P* = 0.0004). The E-IF% of L_4_/_5_ decreased from 89.6% ± 1.9 to 88.2% ± 1.7% after LCT, with an average reduction of 1.66%. The paired *t*-test indicated no significant difference in this index (*t =* 2.553, *P* = 0.0511, Table 3). After the analysis of the main effect variance, no significant differences were noted in the E-IF% of L_3_/_4_ and L_4_/_5_ between the two groups, regardless of whether they were grouped by load weight or load time (Table 4).

### Lumbar curvature index

The LSA increased from 26.622° ± 5.303° to 32.422° ± 6.816° after LCT, with an average increase of 21.79%. The paired *t*-test showed a significant difference in LSA (*t =* − 4.911, *P* = 0.0012, Table 3). The LLA increased from 24.011° ± 7.117° to 30.644° ± 7.415° after LCT, with an average increase of 27.63%. The paired *t*-test indicated a significant difference (*t =* − 4.851, *P* = 0.0013, Table 3).

The results of the main effect variance analysis showed no significant difference between the groups with different load times. However, a significant difference in the LSA was noted among different load weight groups (*F* = 8.228, *P* = 0.0191), while the LLA showed no significant difference (Table 4).

### Questionnaire results

The NRS score results showed that eight soldiers had mild back pain (NRS score from 1 to 3) after LCT, while only one soldier suffered moderate back pain (NRS score 5). The fatigue degree score results indicated that all soldiers performed intense physical activities (fatigue degree score > 6) after LCT, and two of them felt totally exhausted (fatigue degree score 10) (Table 5).

**Table 5:** Results of the questionnaire survey (*n*)

Subject	NRS score of low back pain		Fatigue degree score	
	Pre LCT	Post LCT	Pre LCT	Post LCT
No. 1	0	0	3	9
No. 2	0	0	1	6
No. 3	0	1	2	9
No. 4	0	0	2	7
No. 5	0	0	3	10
No. 6	0	0	1	8
No. 7	0	0	2	8
No. 8	0	1	5	10
No. 9	0	0	2	8

*NRS* Numerical rating scales, *LCT* Load carriage training

### Prediction model for spinal injury

To establish a prediction model of spinal injury for LCT, we selected the FCSA which had a significant difference and a certain clinical significance to fit the multiple linear regression equation. The result was as follows:

As regards the load weight, the results of the Pearson correlation coefficient analysis showed that the correlation coefficient between the △FCSA% and load weight was 0.761, which was a high value (close to 0.8), indicating that the correlation is strong, and a linear relationship can be established. With regard to the load time, the correlation coefficients of △FCSA% was − 0.176, and the relative value was low (< 0.6), suggesting the absence of a linear relationship.

**Fig. 4:**
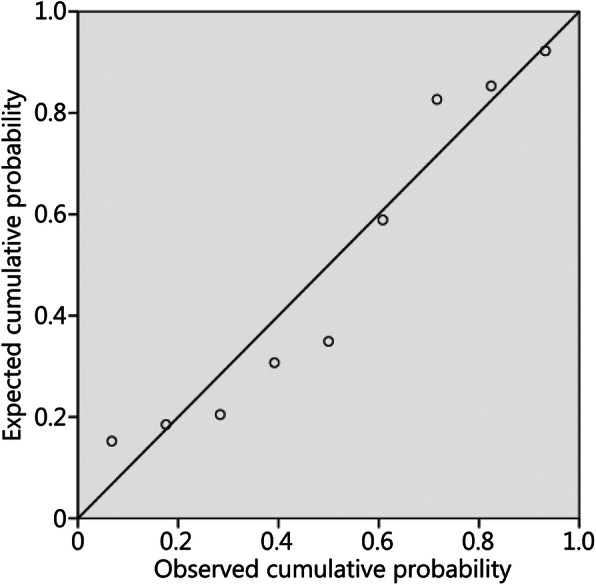
Normal p-p plot of regression standardized residual

To fit the multiple linear regression equation of △FCSA%, the load weight value was introduced into the prediction model as a variable; the result of the analysis of variance of the multiple regression indicated that the *F*-value of this model was 9.612 with a *P*-value of 0.017 (< 0.1), indicating a linear relationship between △FCSA% and load weight. In the regression coefficient analysis, the constant term was − 0.718, and the partial regression coefficient b1 was 23.085 after the *t*-test (*P* = 0.017 (< 0.1)). The regression coefficient of load time outside the equation of the model was re-tested, and its *P*-value was 0.515 (> 0.1) which could not be introduced. The maximum absolute value of the standardized residual of this model was 1.422 (< 3). The normal p-p plot of regression standardized residual showed that the scattered points were basically distributed on the diagonal, which indicated that the residuals could be judged to be normally distributed (Fig. 4). Finally, we obtained the multiple linear regression equation, △FCSA% = − 0.718 + 23.085 × load weight, which can be used to describe the influence of load weight on the change rate of FCSA during LCT.

## Discussion

### Changes to the spine system

To compensate for plateau LCT, certain anatomical or physiological changes will occur in the lumbar spine system. The results showed that both CSA and FCSA had an increasing trend after LCT. However, no significant change was noted in the FCSA/CSA after LCT, proving that the proportion of the lean muscle mass remained basically unchanged after acute high-intensity loading and confirming that the mass and volume of the muscle fibers did not increase after short-term LCT according to the imaging study [29, 30]. That finding indicates that the volume of the paraspinal muscle tissue increased after loading, which may be clinically related to some physiological changes such as muscle congestion and tissue edema. Similarly, the gray value of the paraspinal muscles decreased significantly after LCT, indicating that the mass of the muscle fibers did not increase again. The lumbar paraspinal muscles function by resisting load, stretching or flexing the spine, maintaining the stability of the spine joints, and protecting the discs and ligaments from excessive strain [31]. Excessive LCT with long duration and short rest can lead to aseptic inflammatory reactions, such as muscle cell necrosis, muscle fiber rupture, tissue fluid exudation, and lactic acid accumulation, that result in training injuries of the paraspinal muscles [32].

In terms of lumbar IVDs, the results showed that the total height of the discs decreased after LCT, and the paired *t*-test results showed a significant difference in the change in the IVD height, which proved that the entire spinal system was compressed after loading and the IVD provided a certain cushioning effect. However, in view of clinical medicine, IVDs generally provide the secondary support to the spine during weight loading. The specific mechanism involves sensing the position of the vertebral body through nerve feedback to activate the contraction of the paraspinal muscles during movement and accomplishes stabilization and support of the articular structures [33, 34]. From an anatomical perspective, the bone, ligaments, and other tissues around the IVD have certain limitations on its deformation. Therefore, the height change of the IVD is limited under the loading state that the body can accept. Meanwhile, LCT significantly increases repetitive cycling pressure on the IVD, and the force perpendicular to the ground is also transmitted to the IVD. These mechanical factors eventually cause damage to the IVD [35].

Regarding the lumbar intervertebral foramen, the results showed that the E-IF% in the L_3_/_4_ and L_4_/_5_ levels decreased after LCT. From an anatomical view, when pathophysiological changes such as disc herniation, facet joint swelling, synovial swelling, or bony hyperplasia occur, foraminal stenosis may develop. When the E-IF is less than the diameter of the nerve root, nerve root compression occurs and manifested as pain in the lower back and buttocks and radiating pain or numbness in the limbs; this is an important mechanism for the development of lumbar spine diseases [36, 37]. Mayoux-Benhamou et al. [38] suggested that when the height of the IVD is reduced to 4 mm, that is, 35–50% of the normal height, the reduction in the foraminal area causes nerve compression. However, our results showed a 1-mm reduction in IVD after LCT, which cannot cause compression. Moreover, there is a known correlation between the IVD and the narrowest area of the neuroforamen (*R*^2^ = 0.56) [39]. Our study confirmed that for soldiers without previous spinal degenerative disease, posterior IVD herniation generated by LCT will occupy the available space of the intervertebral foramina and even cause irreversible lesions in severe cases, which is an important pathogenesis of training-related injury symptoms.

As for the lumbar curvature, the LLA increased by 27.63%, while the LSA increased by 21.79% after LCT in the midsagittal image, both showing an increasing trend and having significant differences after LCT. Anatomically, the LLA and LSA can indirectly reflect the stability of the lumbar spine [40, 41]. If the LLA or LSA is too large, it will affects the balance of the spinal system, increases the load on structures such as the paraspinal muscles and IVDs, and increases the probability of injury [42]. The increases in these two angles confirmed the compensatory effect of the lumbar curvature on weight loading. However, the variation in the lumbar curvature was confirmed that it was not the main compensatory mechanism for loading and had a certain upper limit. Moreover, the comparison results of the LSA between groups with different load weights showed significant differences, confirming that the change rate of the LSA has a certain correlation with the load weight that has the potential to become an evaluation indicator for LCT.

In accordance with the questionnaire results, although we found that all soldiers suffered varying degrees of low back pain after LCT, most of them just experienced mild pain and only one soldier experienced moderate pain [43]. These back pain symptoms related to LCT could be relieved by rest. Moreover, the fatigue degree score showed that all soldiers performed intense physical activities during LCT, which indicated a high intensity of the current training. In addition, none of soldiers had other physical symptoms after LCT. Beyond that, the radiologists reviewed all MRI scans after examination and did not find evidence of spinal injury. These results confirmed that the current LCT with different experimental conditions achieved desirable training effect without causing acute irreversible injury to the lumbar spine in this study.

### Prediction model

The linear regression equation of the change rate, △FCSA% = − 0.718 + 23.085 × load weight, was successfully established as a predictive model of soldiers’ spinal injury after plateau LCT. According to this equation, we believe that as the load weight increases, the △FCSA% will increase gradually, and the FCSA value will present an increasing trend. Therefore, we can use this prediction model to estimate that when the load weight reaches 100% of the soldier’s weight, the FCSA is expected to increase by approximately 22.367% after LCT. Since FCSA represents the lean muscle volume within the paraspinal muscle fascia, the variation trend of the prediction model further confirmed that plateau LCT can induce a certain proportion of hypertrophy and proliferation of muscle fiber cells. However, according to the physiological structure of the paraspinal muscle, the FCSA cannot increase indefinitely, and both the CSA and FCSA will eventually reach a peak with the increase in training intensity. Therefore, as the load weight continues to increase, we speculate that the partial regression coefficient b1 (23.085) of this prediction model will gradually decrease and the constant term (− 0.718) will gradually increase; finally, the △FCSA% will reach the upper limit, that is, the FCSA will reach the maximum threshold. However, this prediction model has not yet covered this range.

According to the results of questionnaire survey and radiologists review, it can be concluded that when the maximum load weight does not exceed 60% of their weight, soldiers will not experience paraspinal muscle injury related to short-term LCT, proving that a load weight < 60% is within the tolerable load range of the lumbar paraspinal muscles. As a consequence, this prediction model can be applied to the current conventional weight-bearing training for soldiers in the plateau (the proportion of weight-bearing is less than 60%), which can provide a reference for the formulation of the load weight standard for plateau soldiers under training or combat and improve the scientificity and safety of LCT in the plateau environment.

### Limitations

This study performed actual human experiments in plateau troops for LCT. Results should be interpreted in consideration of the limitations. First, due to military requirements and ethical principles, this experiment could not recruit a sufficient number of subjects. Although a uniform experimental design was used, it cannot completely offset the bias of test data and differences between subjects caused by the small sample size. We hope to recruit more subjects in subsequent studies to verify the present results and obtain further physiological changes in the spine system following plateau LCT. Second, due to ethical restrictions, we were unable to conduct LCT on soldiers using a load weight > 60% or a load time longer than 60 min at present; therefore, we could not directly determine the load weight and load time that may cause physiological damage. Thus, our prediction model also cannot cover the threshold range. We will further validate and optimize this model in subsequent experiments and estimate the training threshold through a more accurate mathematical model.

## Conclusion

This study directly confirmed that the spinal system encountered the following changes after short-term and high-load weight training: increased muscle volume, muscle congestion, tissue edema, IVD compression, decreased E-IF area, increased lumbar curvature, and other physiological changes that reveal important pathophysiological mechanisms of lumbar spinal disorders in soldiers. Moreover, this study established an injury prediction model of the spinal system for plateau LCT through multiple regression analysis: △FCSA% = − 0.718 + 23.085 × load weight. Combined with the results of the questionnaire survey and the radiologists review, this model confirmed that a load weight < 60% of soldiers’ weight cannot cause acute pathological injury in short-term LCT, providing a reference supporting the formulation of the load weight standard for the LCT and, thus, improving the scientificity and safety of military LCT in plateau environments. In addition, the simulated weight load device, image processing methods, and regression analysis method involved in this research have certain universality that can be applied to clinical research of spinal diseases and have considerable application prospects.

## Data Availability

The datasets generated and/or analyzed during the current study are not publicly available due to the individual privacy, but are available from the corresponding author on reasonable request.

## Abbreviations

CSA: Cross-sectional area
E-IF%: Percentage of effective intervertebral foramen area
E-IF: Effective intervertebral foraminal
FSCA: Functional cross-sectional area
IF: Intervertebral foraminal
IVD: Intervertebral disc
LCT: Load carriage training
LLA: Lumbar lordosis angle
LSA: Lumbosacral angle
MRI: Magnetic resonance imaging
US: United States
